# Analysis of novel Sjogren’s syndrome autoantibodies in patients with dry eyes

**DOI:** 10.1186/s12886-017-0412-8

**Published:** 2017-03-07

**Authors:** Sandra Everett, Sahana Vishwanath, Vanessa Cavero, Long Shen, Lakshmanan Suresh, Kishore Malyavantham, Norah Lincoff-Cohen, Julian L. Ambrus

**Affiliations:** 1 Department of Ophthalmology, SUNY at Buffalo School of Medicine, Buffalo, NY USA; 2 Department of Medicine, SUNY at Buffalo School of Medicine, Buffalo, NY USA; 3 Dental School, SUNY at Buffalo School of Medicine, Buffalo, NY USA; 4 Immco Diagnostics, Buffalo, NY USA; 5 Department of Neurology, SUNY at Buffalo School of Medicine, Buffalo, NY USA; 6 Division of Allergy, Immunology and Rheumatology, SUNY at Buffalo School of Medicine, Room C281, Buffalo General Hospital, 100 High Street, Buffalo, NY 14203 USA

**Keywords:** Sjogren’s syndrome, Dry eyes, Corneal abrasion, Autoantibodies

## Abstract

**Background:**

Dry eye is a common problem in Ophthalmology and may occur for many reasons including Sjogren’s syndrome (SS). Recent studies have identified autoantibodies, anti-salivary gland protein 1 (SP1), anti-carbonic anhydrase 6 (CA6) and anti-parotid secretory protein (PSP), which occur early in the course of SS.

The current studies were designed to evaluate how many patients with idiopathic dry eye and no evidence of systemic diseases from a dry eye practice have these autoantibodies.

**Methods:**

Patients from a dry eye clinic and normal controls were assessed by Schirmer’s test for tear flow. Sera were assessed for autoantibodies using ELISA assays. Statistics was performed with Prism 7 software and student’s unpaired *t* test.

**Results:**

In this study 60% of the dry eye patients expressed one of these autoantibodies. Only 30% expressed one of the autoantibodies associated with long-standing SS, which are included in the diagnostic criteria for SS, anti-Ro and anti-La. Patients with disease for less than 2 years and mild dry eyes did not express anti-Ro or anti-La, while 25% expressed anti-SP1. Similar observations, with smaller numbers, were made when patients had not only dry eye but also dry mouth.

**Conclusions:**

Antibodies to SP1, CA6 and PSP occur in some patients with idiopathic dry eyes. Further studies will be needed to determine how many of these patients go on to develop systemic manifestations of SS. Testing for these autoantibodies may allow early recognition of patients with SS. This will lead to improved management of the patients and the development of new strategies to maintain normal lacrimal and salivary gland function in patients with SS.

## Background

Dry eyes and their complications are commonly seen in Ophthalmology practices [1, 2]. Recent studies have demonstrated that Sjogren’s syndrome (SS) is common among patients with dry eyes and is likely underestimated in this patient population [3, 4]. Current diagnostic criteria for SS include serological tests, anti-nuclear antibodies (ANA), rheumatoid factor (RF), anti-La and anti-Ro [5]. Studies investigating dry eye patients with SS diagnosed by clinical criteria note that anti-Ro and anti-La may frequently be negative [6]. Lip biopsies are then required to make a diagnosis of SS.

Studies initially utilizing animal models for SS and various subgroups of SS patients have demonstrated novel autoantibodies, anti-salivary gland protein 1 (SP1), anti-carbonic anhydrase 6 (CA6) and anti-parotid secretory protein (PSP) [7]. In animal models, anti-SP1, anti-CA6 and anti-PSP are expressed earlier in the course of the disease than anti-Ro and anti-La. Various studies have suggested that this may be true in patients with SS [7, 8].

The current studies were established to investigate the presence of anti-SP1, anti-CA6 and anti-PSP in a population of patients with dry eyes. Patients were classified according to the degree of dysfunction noted by Schirmer’s test. No patients had significant medical co-morbidities. Normal controls were obtained from the general community.

## Methods

### Study design

This was a retrospective study performed on a population of patients being followed in a Dry Eye Practice (Investigator SE). All patients were included in the study who lacked a primary cause for their dry eyes, such as medication with anti-cholinergic effects, radiation therapy, sarcoidosis, hepatitis C, HIV, lymphoma or graft-versus-host disease. Normal controls were obtained from the general population. The Institutional Review Board, SUNY at Buffalo School of Medicine, approved these studies.

### Clinical studies

Schirmer’s tests were performed as previously described [9]. Tear osmolarity was determined by The TearLab Osmolarity System following the manufacturer’s instruction. The serology studies for anti-Ro (IgG), anti-La (IgG), anti-SP1 (IgG, IgA & IgM), anti-CA6 (IgG, IgA & IgM), and anti-PSP (IgG, IgA & IgM), were performed at by utilizing enzyme linked immunosorbent assay (ELISA) at Immco Diagnostics, Buffalo, NY [10].

### Statistics

Statistical analysis was performed using Prism 7 software. Comparison of various groups was performed using unpaired student’s *t* test.

## Results

The characteristics of the patients studied are shown in Table 1. The patients with Schirmer’s tests < 3 mm had a longer duration of disease than the patients with Schirmer’s tests 3 mm < SCH < 6 mm, but interestingly both groups had similar ages. The majority of patients in each group had only dry eyes, but the presence of dry mouth was present in similar numbers in each group. Of the patients with Schirmer’s test < 3 mm, one had rheumatoid arthritis and one had diabetes and hypertension. Of the patients with Schirmer’s tests 3 mm < SCH < 6 mm, two had diabetes, four had hypertension and two had allergic rhinitis. The remainder of the patients lacked other significant medical conditions.

**Table 1:** Patient Characteristics

	SCH < 3 mm	3 mm < SCH < 6 mm	Normal
Number in Group	27	38	35
Age	40 – 95 (mean 67.8) yrs.	44 – 80 (mean 63.8) yrs.	31-93 (mean 57.4 years)
Duration of Dry Eyes	1 – 30 (mean 13) yrs.	0.5 – 10 (mean 4.1) years	None
Presence of Dry Mouth	9 patients	8 patients	None

**Fig. 1:**
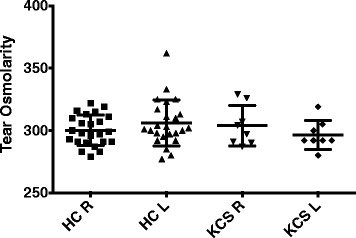
Tear osmolarity studies were done as described in materials and methods. Data shown are the tear osmolarity in the right and left eye of the normal controls (HC) and the patients with keratoconjunctivitis sicca (KCS)

Because the use of tear osmolarity has been suggested as a good indicator for lacrimal gland dysfunction in early Sjogren’s syndrome [11], we evaluated tear osmolarity in normal controls and patients with keratoconjunctivitis sicca. Figure 1 demonstrates that tear osmolarity did not distinguish these two groups (*p* = .388). We did not therefore use tear osmolarity further in our studies.

**Fig. 2:**
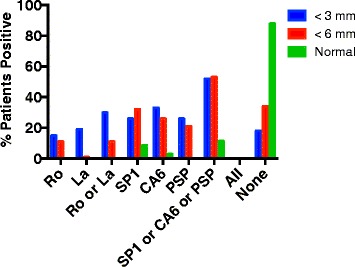
Antibodies to Ro, La, SP1, CA6 and PSP were determined by ELISA in the sera of patients with idiopathic dry eyes and Schirmer’s tests < 3 mm (27 patients), Schirmer’s tests < 6 mm (38 patients) or normal controls (35 patients). Data shown are the percent positive in each group for each of these autoantibodies

The evaluation of autoantibodies in these patients led to very interesting results. As shown in Fig. 2, the novel autoantibodies, anti-SP1, anti-CA6 and anti-PSP were present equally in both groups of patients, those with Schirmer’s tests 3 mm < SCH < 6 mm and those with Schirmer’s tests < 3 mm. In the patients with Schirmer’s tests < 3 mm, the difference between expression of Ro/La versus SP1/CA6/PSP was not statistically significant (*p* = .175). The difference in Ro/La expression (*p* = .036) or SP1/CA6/PSP (*P* < .003) between the patients with Schirmer’s < 3 mm compared to the normal controls was significant. When looking at the patients with Schirmer’s tests 3 mm < SCH < 6 mm, the difference in expression of SP1/CA6/PSP was greater than the expression of Ro/L a in the patients (*p* <. 001) and greater in the patients than in the normal controls (*p* = .0001). The difference in Ro/La expression in these patients was not significantly different than the expression of Ro/La in the normal controls (*p* = .93). Anti-Ro and anti-La antibodies were more common in the sera of patients with less tear production.

**Fig. 3:**
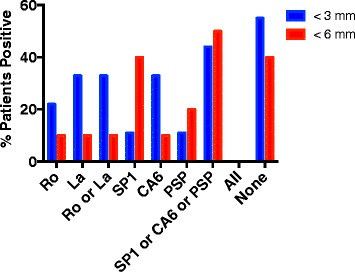
Antibodies to Ro, La, SP1, CA6 and PSP were determined by ELISA in the sera of patients with idiopathic dry eyes and dry mouth with Schirmer’s tests < 3 mm (9 patients), Schirmer’s tests < 6 mm (10 patients). Data shown are the percent positive in each group for each of these autoantibodies

Figure 3 demonstrates that similar findings were observed when the analysis included only those patients with both dry eyes and dry mouth. When comparing the expression of anti-SP1 with that of anti-CA6 and anti-PSP a few observations were notable. In patients with dry eyes and dry mouth and Schirmer’s tests 3 mm < SCH < 6 mm, anti-SP1 was the dominant autoantibody while in the patients with Schirmer’s tests < 3 mm, anti-CA6 was the dominant autoantibody. However, the numbers were small so that no conclusions can be drawn from these results. In patients with only dry eyes, equal numbers of patients express either anti-SP1, anti-CA6 or anti-PSP with several patients expressing two or all three of these autoantibodies.

**Fig. 4:**
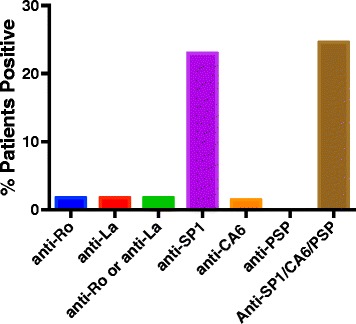
Antibodies to Ro, La, SP1, CA6 and PSP were determined by ELISA in the sera of 24 patients with idiopathic dry eyes for less than 2 years and Schirmer’s tests < 6 mm. Data shown are the percent positive for each of these autoantibodies

To further evaluate patients with very early disease, we examined the autoantibody studies in 24 patients in the cohort who not only had Schirmer’s test 3 mm < SCH < 6 mm, but also had symptomatic dry eyes for less than 2 years. None of these patients had dry mouth. As shown in Fig. 4, this group did not express anti-Ro or anti-La. Anti-SP1 antibodies were seen in 25% of the patients. The difference in expression of anti-Ro/anti-La compared to anti-SP1/anti-PSP/anti-CA6 is statistically significant (*p* = .0081).

**Fig. 5:**
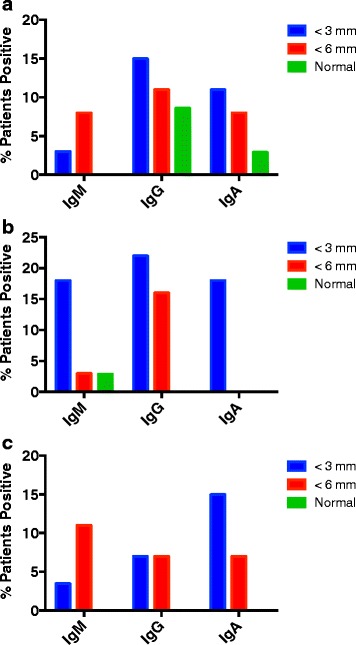
**a** IgM, IgG and IgA autoantibodies to SP1 were determined by ELISA in the sera of patients with idiopathic dry eyes and Schirmer’s tests < 3 mm (27 patients), Schirmer’s tests < 6 mm (38 patients) or normal controls (35 patients). Data shown are the percent positive in each group for each of these autoantibodies. **b** IgM, IgG and IgA autoantibodies to CA6 were determined by ELISA in the sera of patients with idiopathic dry eyes and Schirmer’s tests < 3 mm (27 patients), Schirmer’s tests < 6 mm (38 patients) or normal controls (35 patients). Data shown are the percent positive in each group for each of these autoantibodies. **c** IgM, IgG and IgA autoantibodies to PSP were determined by ELISA in the sera of patients with idiopathic dry eyes and Schirmer’s tests < 3 mm (27 patients), Schirmer’s tests < 6 mm (38 patients) or normal controls (35 patients). Data shown are the percent positive in each group for each of these autoantibodies

In animal models of SS, the autoantibodies identified early in the course of the disease are IgM antibodies [12]. At later stages of the disease, IgA and IgG antibodies are noted. We therefore examined the isotypes of the anti-SP1, anti-CA6 and anti-PSP antibodies in the patients evaluated in these studies. Figure 5a evaluates anti-SP1. There is a tendency in the patients with Schirmer’s <6 mm to have more IgM antibodies, but because the numbers are small, none of the differences seen are statistically significant. Figure 5b evaluates anti-CA6 antibodies. Patients with Schirmer’s <3 mm had more IgM anti-CA6 than both patients with Schirmer’s 3 mm < SCH < 6 mm (*p* = .02) and normals (*p* = .02). With the IgG anti-CA6 both the Schirmer’s < 3 mm (*p* = .002) and the Schirmer’s 3 mm < SCH < 6 mm (*p* = .02) patients were significantly greater than the normal controls. The IgA anti-CA6 were similar to the IgM anti-CA6 in that the Schirmer’s < 3 mm had more antibody than both the patients with Schirmer’s 3 mm < SCH < 6 mm (*p* = .005) and the normal controls (*p* = .005). Fig. 5c evaluates anti-PSP antibodies. In these studies, because of the small numbers, the only statistically significant changes were IgM anti-PSP Schirmer’s 3 mm < SCH < 6 mm versus normal controls (*p* = .04) and IgA anti-PSP Schirmer’s < 3 mm versus normals (*p* = .01). This suggests that even the patients that are picked up at what would be an early stage of SS, i.e. without systemic manifestations, have in fact had their autoimmune eye disease for a long period of time. Interestingly, patients with Schirmer’s tests 3 mm < SCH < 6 mm had symptoms of dry eyes for an average of 4.1 years while those with Schirmer’s tests <3 mm had symptoms of dry eyes for an average of 13 years. It has been noted in several studies that identification of patients with early SS is a major challenge [13].

## Discussion

The current studies demonstrate that many patients with “idiopathic” dry eyes, with or without dry mouth, have autoantibodies that are seen in patients with early SS. Some of these patients have autoantibodies included in the current diagnostic criteria for SS, anti-Ro and anti-La, while more patients express more recently discovered autoantibodies associated with an early stage of SS, anti-SP1, anti-CA6 and anti-PSP, without anti-Ro or anti-La.

Previous studies from an academic Sjogren’s center demonstrated that many patients referred with a diagnosis of dry eyes in fact had SS that met full criteria established by the American College of Rheumatology [4]. Many of these patients had evidence of systemic manifestations of SS as well. In the current study, patients were studied from a private dry eye practice who lacked evidence of systemic manifestations of SS. These patients were in fact often self-referred or referred by an Internist for an initial evaluation. They likely had earlier disease than the patients referred to a tertiary academic center. Nonetheless, there was still a significant delay in the evaluation by the Ophthalmologist relative to the onset of dry eyes as indicated by the clinical history but also by the fact that the predominant autoantibodies were of the IgG or IgA isotype rather than the IgM isotype.

Animal models have demonstrated that the earliest manifestations of SS are loss of salivary gland function secondary to involvement of the innate immune system [7, 12, 14]. Lacrimal gland involvement likely occurs initially in the same manner, although this has not yet been studied completely. At this stage, antibodies anti-SP1, anti-CA6 and anti-PSP are noted. In the next stage lymphocytic infiltration of the glands occurs followed by progression from a local disease to a systemic disease, with lung and kidney involvement. Antibodies anti-Ro and anti-La are identified in the animals at the point when the disease goes from being local to being systemic [7]. It should be noted, however, that one study demonstrated anti-Ro and anti-La antibodies in the sera of patients who developed SS years before clinical expression of their disease [15, 16]. So, in some cases, anti-Ro and anti-La may occur in early disease as well. The challenge from a therapeutic point of view is to identify the SS patients at the very earliest stages, before there has been permanent damage to the lacrimal and salivary glands [17–19]. In animal models, treatment at this stage can lead to halting of the disease process [20–22]. The current studies suggest that Ophthalmologists see patients who are in very early stages of SS. Perhaps with greater vigilance in the medical community, Ophthalmologists can see these patients at even earlier stages.

At the same time, many of the patients in this study had dry eyes for longer than 10 years without the development of systemic symptoms. They had autoantibodies consistent with immune mediated lacrimal gland injury. Perhaps many patients develop a form of SS that involves only the lacrimal and/or salivary glands without ever progressing to more systemic manifestations. These patients would not meet the accepted criteria for SS, but in fact may deserve the same type of immunomodulatory therapy. This therapy could theoretically be given locally under these circumstances rather than systemically. Further study will be necessary to address these issues.

In conclusion, many patients with “idiopathic dry eyes” identified in a private dry eyes practice have evidence of immune mediated lacrimal gland injury with serum autoantibodies consistent with early SS. The criteria for SS may have to change to accommodate these patients. Future studies will be necessary to determine the most appropriate therapies for these patients.

## Conclusions

The current studies demonstrate that many patients with idiopathic dry eyes with or with out dry mouth have markers for early Sjogren’s syndrome, anti-SP1, anti-CA6 and anti-PSP. Further study will be necessary to assess antibody positive patients for various features of SS over a long period of time and to develop therapies appropriate for patients showing evidence of early SS.

## Abbreviations

ANA: Antinuclear antibodies
CA6: Carbonic anhydrase 6
ELISA: Enzyme linked immunosorbent assay
PSP: Parotid secretory protein
RF: Rheumatoid factor
SP1: Salivary gland protein 1
SS: Sjogren’s syndrome
